# Emerging Approaches for Regulation and Control of CAR T Cells: A Mini Review

**DOI:** 10.3389/fimmu.2020.00326

**Published:** 2020-02-26

**Authors:** Lærke J. B. Brandt, Mike B. Barnkob, Yale S. Michaels, Julia Heiselberg, Torben Barington

**Affiliations:** ^1^Department of Clinical Immunology, Odense University Hospital, University of Southern Denmark, Odense, Denmark; ^2^School of Biomedical Engineering, University of British Columbia, Vancouver, BC, Canada

**Keywords:** chimeric antigen receptor, cancer, immunotherapy, T cell, synthetic, regulation, cell therapy

## Abstract

Chimeric antigen receptor (CAR) T cells have emerged as a promising treatment for patients with advanced B-cell cancers. However, widespread application of the therapy is currently limited by potentially life-threatening toxicities due to a lack of control of the highly potent transfused cells. Researchers have therefore developed several regulatory mechanisms in order to control CAR T cells *in vivo*. Clinical adoption of these control systems will depend on several factors, including the need for temporal and spatial control, the immunogenicity of the requisite components as well as whether the system allows reversible control or induces permanent elimination. Here we describe currently available and emerging control methods and review their function, advantages, and limitations.

## Introduction

Chimeric antigen receptor (CAR) T cells have emerged as a promising treatment for patients with advanced B-cell cancers (1–3) but more effective control of the therapy is needed to combat associated toxicity and to expand CAR therapy toward other cancer types. CAR T cells are a personalized immunotherapy, in which allogeneic or autologous T cells are genetically modified to express a synthetic construct, combining an extracellular binding domain, often an antibody-derived single chain variable fragment (scFv), with activating signaling domains from the T-cell-receptor complex, such as CD3ζ, CD28, and 4-1BB. Recognition of cell-surface proteins through the extracellular domain allows CAR T cells to target cancer cells for cytotoxic killing (4).

As a living drug, CAR T cells bear the potential for rapid and massive activation and proliferation, which contributes to their therapeutic efficacy but simultaneously underlies the side effects associated with CAR T-cell therapy. The most well-known toxicity is called cytokine release syndrome (CRS) which is a systemic inflammatory response characterized by fever, hypotension and hypoxia (5–7). CRS is triggered by the activation of CAR T cells and their subsequent production of pro-inflammatory cytokines including IFNγ, IL-6 and IL-2 (8). This is thought to result in additional activation of bystander immune and non-immune cells which further produce cytokines, including IL-10, IL-6, and IL-1 (9). The severity of CRS is associated with tumor burden, and ranges from a mild fever to life-threatening organ failure (10, 11). Neurologic toxicity is another serious adverse event which can occur alongside CRS (12). Although the pathomechanism is unknown, it is believed to be the result of cerebral endothelial dysfunction (13). Finally, since few antigens are truly tumor specific, toxicities can arise if CAR T cells target healthy cells expressing the recognized antigen i.e., on-target, off-tumor activity. Unfortunately, this has led to severe and fatal outcomes, especially when targeting antigens in solid tumors, hampering CAR T-cell application in these patients (14–17).

Current clinically approved CAR designs do not enable control over CAR T cells following infusion, and so management of toxicities depends on immuno-suppression using systemic corticosteroids as well as an IL-6 receptor antibody, tocilizumab. Unfortunately, the use of immunosuppressive drugs severely limits the time span CAR T cells are functional (11). Given the severity of the toxicities, as well as the manufacturing costs, there is a clinical need to regulate CAR T-cell numbers and activity once deployed in patients. In this mini review, we describe existing and emerging approaches to regulation and control of CAR T cells, and discuss each method's advantages and disadvantages.

## Passive Control

**Figure 1:**
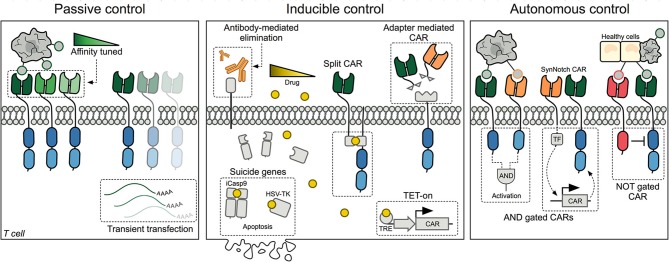
Schematic representation of the three major methods designed for controlling CAR T cells today. **Left panel:** Passive control methods include affinity tuned CARs and transient transfection of T cells. **Middle panel:** Inducible control includes methods to eliminate CAR T cells using antibodies or inducible suicide systems. Additionally, different drugs have been utilized to either control CAR expression at the transcriptional level or assembling of a split-CAR, where the extra- and intracellular domains have been separated. Another approach has been to decouple the binding domain from the intracellular signaling domain, such that binding adapters can be supplied and titrated. **Right panel:** Autonomous CAR T cells are self-regulated and can decide whether to initiate or withhold cytotoxic killing of target cells based on surface proteins expressed by healthy and cancerous cells. CAR, Chimeric Antigen Receptor; TRE, Tetracycline Response Element; TF, Transcription Factor; SynNotch, Synthetic Notch receptor.

Passive control methods provide straightforward opportunities to limit CAR T-cell mediated cytotoxicity, but offer no downstream control over engrafted cells following transfusion (Figure 1, left panel).

### Transient Transfection

A simple but effective way of regulating CAR T cells consists of transiently transfecting T cells with CAR-encoding mRNA (18–23). Due to the lack of genomic integration, CAR expression is limited by the degradation of the CAR-encoding mRNA and dilution following each T-cell division (18). The result is a steady decrease in CAR-expressing T-cell numbers, unless new cells are infused. Repeated infusions are however associated with a higher risk of an anaphylactic reaction due to the CAR T cells (24). While the inherently limited persistence of these CAR T cells might compromise continued anti-leukemic effect (25), it also limits long-term hematologic toxicities and off-target effects.

### Affinity Tuning

Lowering the binding domain's affinity toward the targeted antigen aims to prevent on-target, off-tumor toxicities from arising in the first place (26, 27). While affinity-tuned CARs retain the ability to bind to cancer cells with a high antigen expression, healthy tissues with lower expression are spared (28). The use of low-affinity CARs is therefore especially interesting when targeting antigens known to be expressed on healthy tissue in low amounts, e.g., HER2 or EGFR (26, 27). This, however, might also lead to cancer cell escape variants with low antigen expression (29). Additionally, both promoter usage and transduction level of T cells might result in heterogeneous expression of the CAR protein, making it hard to ensure consistent behavior among individual CAR T cells as their avidity toward the antigen can vary. One promising strategy to overcome heterogeneous CAR expression is to instead integrate the CAR construct into the endogenous TCR alpha chain (TRAC) locus using the CRISPR/Cas9 system (30).

## Inducible Control

Recognizing that CAR T-cell toxicities arise rapidly, researchers have developed several exogenous methods to quickly regulate the activity of the CAR T cells or to eliminate them completely. These methods rely on co-administration of a drug, thereby making their use dependent on pharmacokinetics, tissue availability, and potential adverse effects of the chosen drug (Figure 1, middle panel).

### Suicide Genes

Depletion of CAR T cells can be achieved by designing CAR constructs that also express a suicide gene, such as inducible Caspase 9 (iCasp9) (31–39), herpes simplex virus tyrosine kinase (HSV-TK) (40, 41) or human thymidylate kinase (TMPK) (42). In cells expressing iCasp9 and TMPK, elimination is achieved through activation of the caspase 3 apoptotic pathway when a small molecule is administered. The iCasp9 system has successfully been validated in patients receiving haploidentical stem-cell transplants (HSCT) in which iCasp9-expressing T cells were rapidly removed at onset of graft-versus-host disease (GvHD) (31). Likewise, administration of ganciclovir to T cells co-expressing HSV-TK causes formation of a toxic metabolite but cell death may take up to several days as it depends on cell proliferation (40, 41). The use of HSV-TK is severely limited by the high immunogenicity of the virally-derived protein (43). Furthermore, the HSV-TK suicide system is complicated by the fact that ganciclovir is used as a first-line treatment against cytomegalovirus (CMV) infection, a virus which is often reactivated in HSCT and other immunocompromised patients (44). As TMPK and iCasp9 are of human origin, the risk of immunogenicity is low. Indeed long-term engraftment up to several years in patients infused with iCasp9 expressing cells has been reported (45).

### Elimination Markers

Co-expression of a cell-surface elimination marker, not normally present on T cells, allows for antibody-mediated degradation and control of the CAR T cells (22, 46–52). By utilizing clinically approved antibodies, e.g., rituximab targeting CD20 (48–50) or cetuximab targeting EGFR (51, 52), complement- or antibody-dependent cytotoxicity (CDC/ADCC) can be achieved toward the CAR T cells (51). Choosing a marker that is co-expressed on cancer cells, allows this method to create additional tumor killing, with the caveat that further collateral toxicity might arise. Cell-surface markers also allow for positive selection of transduced T cells in the manufacturing process, and subsequent monitoring of CAR T-cell levels *in vivo*. However, the efficacy of the strategy can be compromised by the fact that CDC/ADCC capacity is limited in patients treated with chemotherapy prior to CAR T infusion (53). In addition, antibodies can have limited biodistribution and tissue penetration, especially in poorly vascularized tumors (54). In order to address these problems, researchers have instead created anti-idiotype CARs recognizing murine CD19-specific CARs (55) or incorporated a short peptide epitope, called an E-tag, into the extracellular domain of the CAR, and created anti-E-tag CARs which could then be used to eliminate the anti-tumor CARs (56).

Since the use of suicide genes and elimination markers result in irreversible depletion of this complex treatment, researchers have developed a number of reversible methods to control CAR T cells as well.

### Systemic T-Cell Inhibition

Current methods for controlling CAR T cells include systemic immunosuppressive agents, e.g., corticosteroids (57). The lymphocytotoxic anti-CD52 antibody alemtuzumab, has also been proposed as a method of depleting CD4- and CD123-specific CAR T cells (47, 58), as targeting these proteins might cause hematological aplasia and toxicity. More recently, it was shown that CAR signaling could be inhibited using the tyrosine kinase inhibitor dasatinib. Dasatinib inhibits phosphorylation of lymphocyte-specific protein tyrosine kinase (LCK), a critical component in the T-cell signaling pathway. Preclinical studies suggest that treatment with dasatinib can reversibly inhibit CAR T-cell proliferation and cytokine production without negatively affecting viability (59, 60). Although dasatinib cannot adequately inhibit already activated CAR T cells, limiting its usage against acutely arising CRS or neurotoxicity, the drug was shown to be superior to dexamethasone in inhibiting further activation in a preclinical study (59). Finally, while corticosteroids and alemtuzumab cause widespread inhibition or complete elimination of both CAR T cells and healthy lymphocytes, dasatinib has the advantage of acting as a faster on/off switch, due to its short half-life of 4 h (61).

### Adapter Mediated CARs

Aiming to specifically control CAR T-cell activity toward the antigen, several models of adapter-mediated CARs, also known as universal CARs, have been developed (62–71). A shared feature is their method of tumor recognition, which is achieved by linking an adaptor, a molecule recognized by the CAR, to an antibody or ligand that recognizes the tumor antigen. While current clinically approved CARs are designed to be constitutively active, adapter-dependent CAR T cells can only recognize and kill when the adapter is administered, allowing for titratable and reversible control of the CAR T cells. A major advantage of this approach is the ability to target different antigens without the need to re-engineer and re-transfuse T cells. Adapters have also been designed to redirect anti-CD19-specific CARs to another target, using CD19-fusion proteins, suggesting that adapter proteins might be used with current clinically approved CAR T cells (72). However, large differences in adapter kinetics and subsequent effects on CAR T cells have been reported, probably reflecting differences in models used, affinity differences between the adapter and both target and CAR T cells, and biodistribution of the adapter molecules.

### Split-CARs

Instead of directly regulating CAR binding to antigen, pharmacological inducers can also be used to control the activity of CAR T cells themselves, by splitting the CAR's extracellular antigen-binding domain from its intracellular signaling domains (73, 74). Assembling of the fully functional CAR is therefore dependent on administration of a dimerizing drug, limiting the CAR activity by the half-life of the drug. Consequently, CAR T-cell activation requires two inputs: the tumor antigen and the dimerizing drug. The split-CAR can thus also be considered an AND gate CAR (see below). The split-CAR allows temporal and reversible control over the number of functional CARs, but the design does not prevent on-target, off-tumor toxicities as no spatial control is achieved due to a lack of control over the distribution of the drug.

### Protease Inhibitors

Strategic incorporation of the autocleaving hepatitis-C-derived NS3 protease in the CAR construct has also been suggested as a way to control CAR T-cell activity. Juillerat et al. incorporated the NS3 protease between the CAR and a degradation moiety, thereby tagging the CAR construct for degradation when a NS3 protease inhibitor was administered (75). This construct showed reversible as well as tunable control over CAR T-cell cytotoxicity *in vitro*. As the NS3 protease is virally derived, it holds immunogenic potential which could potentially limit CAR T-cell persistence.

### TET-On Regulation

It has also been suggested to regulate CARs at the transcriptional level. Several groups have shown that a drug-inducible CAR system can be generated by controlling CAR transcription using the TET-on system, allowing reversible control of CAR T cells (76–78). CAR mRNA is thus only produced in the presence of doxycycline, although some background CAR expression was observed (76). As the TET-on system is derived from both bacteria and virus, it holds significant immunogenic potential with the risk of host mediated elimination of the CAR T cells. Another drawback is the lack of rapid control, should life-threatening side effects occur, due to the control occurring on a transcriptional level. However, for highly proliferative CAR T cells that are constantly diluting the CAR protein amongst daughter cells, transcriptional regulation may be sufficient to limit the quantity of functional CAR complexes.

## Logic Gates and Autonomous Control

As CAR T-cell therapy is applied against solid tumors, the distinction between healthy and malignant tissue becomes increasingly important (79). The use of so-called boolean logic gates and tumor selectivity mechanisms is envisioned to generate autonomous CARs with a higher target specificity, capable of better distinguishing tumor cells from healthy cells (Figure 1, right panel).

### AND Gates

One approach to enable better decision-making in CARs is the incorporation of logic AND gates, such that a combination of antigens are required for activation. Often this dual CAR design consists of two extracellular domains, with specificities toward different antigens, each coupled to separate components of the intracellular stimulatory apparatus, e.g., CD3ζ and CD28 or 4-1BB (80–83). Such approaches have been tested in preclinical prostate and breast cancer models and might allow for targeting of proteins that are also present in healthy tissue (81, 82). A worry, however, is that even partial signaling through one receptor may generate sufficient T-cell activity to cause off-target damage (81, 83). Another approach has therefore been to use one receptor exclusively as a priming signal, with no activating signaling capacity itself. This was achieved using so-called SynNotch receptors, which were coupled to orthogonal transcription factors that were released upon binding. This “priming” leads to expression of a fully functional CAR targeting another cancer-associated antigen, ensuring localized activity of the CAR T cell (84, 85). Importantly, in mouse models the SynNotch CARs did not migrate, but retained their function only in dual positive tumors, indicating that this approach ensured good spatial control of the CAR (84, 85).

### NOT Gates

Better discrimination between malignant and healthy cells can also be achieved by designing an inhibitory CAR (iCAR). The iCAR contains a binding domain specific for an antigen expressed on healthy cells fused to the signaling domains of CTLA-4 or PD-1, such that recognition of the healthy antigen leads to an inhibitory signaling cascade that overrides activating signals by dephosphorylating the receptor complex (86). The iCAR should restrict CAR T-cell activity to tumor tissue lacking the healthy antigen, limiting on-target, off tumor activity. Such a system might allow for CAR T cells previously shown to cause lethal off-tumor activity, such as ERBB2-specific designs, to be re-introduced.

While the AND gate and iCARs restrict CAR T-cells' activity spatially, they cannot control the intensity of the CAR T-cell activity nor control them in a temporal manner.

### Tumor Localizing Mechanisms: Hypoxia Sensitivity and Masked CARs

In order to gain better temporal control and limit CAR T-cell activity to the tumor microenvironment (TME), CAR expression can be controlled by incorporating a hypoxia inducible factor (HIF) in the CAR construct (87). The CAR-HIF construct is continuously targeted for degradation when the CAR T cell is present in a normoxic environment, i.e., most healthy tissues, ensuring that CAR expression only occurs under hypoxic conditions, as seen within parts of the TME. Because degradation of the CAR is regulated at the protein level, control is thought to occur quickly, which is favorable to avoid CAR activity outside of the tumor. This method has only been tested *in vitro*, and may fail to eradicate tumor cells residing in normoxic tissues, e.g., the peripheral parts of the tumor. Moreover, these CAR T cells may show off-target effects in healthy, hypoxic tissues like the bone marrow.

Another method to constrict CAR activity to the TME is by designing a masked CAR, as proposed by Han et al. (88). Here the CARs antigen-binding site is hidden by a masking peptide with a linker sensitive to proteolytic cleavage. Tumor associated proteases present in the TME can then cleave the linker, removing the masking peptide and allowing CAR T cells to target antigen presenting cells. However, a possibility remains that endogenous proteases cleave the masking peptide, paving the way for on-target, off-tumor toxicities.

### CAR T Cells Producing IL-1 Receptor Antagonist (IL-1Ra)

One of the central cytokines involved in CRS is IL-1. Giavridis et al. recently designed a CAR T cell constitutively producing IL-1 receptor antagonist, protecting mice against CRS associated mortality without affecting the anti-tumor efficacy (9). One major advantage of using IL-1 receptor antagonist is its ability to cross the blood-brain barrier, thereby potentially reducing CAR T-cell-related neurotoxicity (89).

While the above mentioned logic gates and hypoxia sensitive CARs seek to enhance tumor specificity, they do not allow the clinicians control over the CAR T cells and as such offer no solution should life-threatening toxicities arise.

## Discussion

The recent years' success of CAR T cells in the clinic has revealed the serious and potentially lethal side effects associated with the potent treatment, including off-tumor, on-target effects, systemic inflammatory conditions such as CRS and acute neurotoxicity (90, 91). More recently it has become clear that cardiovascular and gastrointestinal events can also occur post-CAR-T (92, 93). As the range of specificities and tumor-types targeted using CAR T cells increase, new side-effects will likely come to light. It is therefore important to consider how to best regulate and control engineered T cells.

Because CAR-related toxicities often arise acutely, control mechanisms should ideally grant the clinician swift control over CAR T-cell activity. A direct comparison of the on/off kinetics for each method is made difficult by differences in study design, but regulation on the protein level as well as the use of suicide genes or elimination markers are expected to act faster than regulation at the transcriptional level (Table 1). Permanent elimination of CAR T cells however abrogates the long term anti-leukemic effect and many methods therefore aim at reversible control, allowing the clinician to turn off the CAR T cells when toxicities occur. In the future, an appropriately designed recombinase-mediated switch would allow CAR activity to be stably switched into an OFF state with one small molecule, and subsequently flipped back into an ON state using a second molecule (94). This would give clinicians the power to halt CAR T activity without permanently destroying a costly and life-saving therapeutic product, while avoiding the need to constantly administer the suppressive molecule to maintain an OFF state. Ideally, small molecules that have already gained regulatory approval and show minimal side effects can be co-opted to rapidly and reversibly modulate CAR T-cell activity. The choice of drug however must also be guided by the tumor-type targeted, as endothelial barriers, such as the blood-brain-barrier, and poor vascularization can prevent proper biodistribution and concentration in the effected organs.

**Table 1:** Current approaches to regulation and control of CAR T cells.

	**CAR specific?**	**Default state (On or Off)**	**On/Off kinetics**	**Origin of system**	**State of control (Permanent or reversible)**	**Tested in clinical studies**	**References**
Transient transfection	Yes	ON	11–13 days		Permanent	Phase I NCT03060356 NCT02277522 NCT02624258 NCT02623582	(18–23, 25)
Affinity tuning	Yes	ON			Permanent	Phase I NCT02443831	(26–28)
Suicide genes	Yes	ON	<24 h	Modified human and Viral	Permanent	Phase I/II (iCasp9) NCT03579927 NCT02414269 NCT02107963 NCT01822652	(31–42)
Elimination markers	Yes (not CD52)	ON	<1 h–1 week	Modified human	Permanent	Phase I (RQR8, tEGFR) NCT03590574 NCT02746952 NCT03085173 NCT03618381 NCT02051257 NCT03070327 NCT02028455 NCT02146924 NCT01865617 NCT02937844 NCT03638167 NCT02311621 NCT03114670 NCT02159495	(22, 46–52)
Anti-E-tag CARs	Yes	ON/OFF	>48 h	Murine	Permanent	No	(56)
Dasatinib	No (all T cells)	ON	OFF: 1–2 h ON: 7 h		Reversible	No	(59, 60)
Adapter mediated CARs	Yes	OFF	1 h–11 days	Human or murine	Reversible	No	(62–72)
Split-CARs	Yes	ON	<36 h	Human	Reversible	No	(73, 74)
Protease inhibitors	Yes	ON	<48 h	Viral	Reversible	No	(75)
TET-on regulation	Yes	OFF	12–24 h	Viral and bacterial	Reversible	No	(76–78)
AND gates	Yes	OFF		Synthetic	Reversible	No	(80–83)
SynNotch	Yes	OFF	OFF: 8 h ON: 13 h	Murine	Reversible	No	(84, 85)
NOT gates	Yes	ON (CAR) OFF (iCAR)	<24 h	Human	Reversible	Phase I NCT03824951 NCT02442297	(86)
Hypoxia sensitive CARs	Yes	OFF (normoxia)	6 h	Human	Reversible	No	(87)
Masked CARs	Yes	OFF	<6 h	Bacterial	Reversible	No	(88)
IL-1Ra producing CARs	Yes	ON		Murine		No	(9)

Another approach is to avoid unwanted immune responses from arising at all. This is the rationale behind many of the logic-gated CAR designs developed, including iCARs and tumor-localized CARs (84, 87). Passive and sustained fine-tuning of CAR expression levels could also be achieved by targeted genomic integration (30) or by using degrons (95) or synthetic miRNA regulation (96). Not only does this have the potential to mitigate dangerous cytokine release, reducing CAR expression has also been shown to combat T-cell exhaustion (30).

The adaptation of CARs to recognize and respond to soluble ligands, such as secreted cytokines, were recently reported by Cheng et al. and creates exciting new possibilities in CAR engineering (97). CARs targeting immunosuppressive soluble ligands, such as TGF-beta, could possibly contribute in overcoming the hostile TME, which has proven a major obstacle especially in solid tumors. Likewise, a possible combination of iCARs or SynNotch receptors with the ability to sense inflammatory cytokines could be used to achieve autonomous dynamic feedback control of CAR activity (98). Consequently, this could lead to the creation of CAR T cells capable of responding to heightened levels of inflammatory cytokines, preventing accompanying toxicities. Simultaneously leveraging inducible and autonomous CAR T-cell control methods could substantially improve the safety of CAR therapy. This might be especially useful when targeting solid tumors, where targeted antigens can often be found in other healthy tissues. While autonomous CAR control designs can restrict cytotoxic activity to the time, location and target cells of interest, also including an inducible kill switch will provide an additional fail-safe in the event the engrafted T cells behave unexpectedly or undergo oncogenic transformation.

Early clinical success and challenges have led to an explosion in new technologies for inducibly, autonomously and passively controlling CAR T cell function, providing the community with a growing menu of solutions for safe and effective anti-cancer therapy. Ultimately the desired regulation of CAR T cells will depend on the location, aggressiveness and targetability of the tumor.

## Author Contributions

LB, MB, and YM wrote the manuscript. JH and TB reviewed and edited the manuscript.

### Conflict of Interest

YM and MB have filed a patent relating to a technology presented in this manuscript. The remaining authors declare that the research was conducted in the absence of any commercial or financial relationships that could be construed as a potential conflict of interest.
